# *Brucella*-Induced Acute Psychosis: A Novel Cause of Acute Psychosis

**DOI:** 10.1155/2021/6649717

**Published:** 2021-03-04

**Authors:** Chi-Chi Obuaya, Gayathri Thivyaa Gangatharan, Efthimia Karra

**Affiliations:** ^1^CNWL NHS Foundation Trust, London, UK; ^2^Imperial College London School of Medicine, London, UK; ^3^Physicians' Clinic, London W1G 7AE, UK

## Abstract

**Background:**

Infections have long been linked to psychosis and categorised within “secondary” psychoses. To date, there have been few reports of psychosis linked to brucellosis. This case report aims to present one such case. *Case Presentation*. A 31-year-old man was admitted to a general hospital with pyrexia, severe right upper quadrant pain, and an acute psychosis following a two-week holiday in South East Asia and the Mediterranean. Serological tests revealed that he had brucellosis. Following antibiotic treatment, the psychotic symptoms abated and he was discharged within ten days of hospitalisation.

**Conclusions:**

This case of organic psychosis highlights the importance of considering brucellosis as a rare cause of acute psychosis. The exact mechanism of *Brucella*-induced psychosis remains unclear.

## 1. Background

Psychosis incorporates a loss of touch with reality, accompanied by disordered thoughts and perceptions, including delusions, hallucinations, and confusion; it is sometimes also accompanied by disturbed affect and low mood [1, 2]. Historically, psychotic presentations with a clearly identifiable biological cause, such as poststroke psychosis, have been termed “organic,” and those without an attributable cause, such as schizophrenia, have been termed “functional” psychoses [3].

Over time, it has become apparent that some “functional” psychotic disorders do indeed have underlying pathophysiological causes [4]. The term “primary” has been used to describe psychosis without a known cause and “secondary” when there is an identifiable cause. Psychosis can further be categorised according to the duration of the presentation: acute or transient psychoses last for less than one month.

Causes of secondary psychosis include autoimmune disorders such as N-methyl-D-aspartate receptor encephalitis; congenital disorders, such as agenesis of the corpus callosum; psychoactive substances, including cannabis, LSD (lysergic acid diethylamide), and mercury poisoning; iatrogenic causes, such as steroids or isoniazid; cerebrovascular disorders, including strokes and subdural haematomas; space-occupying disorders; metabolic disorders, such as pheochromocytoma; dietary disorders, such as vitamin D or B12 deficiency; seizure disorders; endocrine disorders, such as hyperthyroidism; degenerative or demyelinating disorders; and infectious disorders [5]. HIV and neurosyphilis remain the most reported infectious causes of psychosis, as well as *Toxoplasma gondil* [6] and *Chlamydia pneumoniae* [7] to a lesser degree.

*Brucella* spp. are zoonotic, Gram-negative, coccobacillus-shaped, facultative aerobes which commonly infect domestic animals (particularly cattle, sheep, goats, and pigs) [8], as well as wildlife and marine animals [9]. Transmission from animals to human is foodborne (often via unpasteurised milk or associated dairy products) or via direct contact (with blood, faeces, urine, or tissues of infected animals) [10]. Rarely, brucellosis can also be acquired via sexual transmission [11]. In cases of laboratory-acquired infection, transmission is due to inhalation of aerosolized particles or inadequate care when handling the infective organism [9].

Brucellosis is commonly diagnosed in the Middle East and North Africa, Mexico, Central and South America, Eastern Europe, the Caribbean, Eastern Mediterranean Region, Central Asia, and the Indian subcontinent [11].

Neurobrucellosis is a rarer manifestation of *Brucella* infection and affects 3–5% of infected patients [9]. It typically presents with diplopia, headaches, coma, paresis, depression, psychosis, and mental fatigue [9, 12].

We identified a limited number of case reports in which psychosis is listed as a symptom of brucellosis [6, 13]. This case report outlines a case of acute psychosis secondary to *Brucella* infection. The possible neurological pathways involved and pathophysiology of neurobrucellosis will be addressed in the discussion part of this report.

## 2. Case Presentation

A 31-year-old man of European origin was admitted to a general medical hospital in London, UK, with pyrexia, right upper quadrant pain, mild constipation, headaches, and night sweats following a two-week holiday in South East Asia and the Mediterranean. The abdominal pain had resided with a low-fat diet, but overall, the symptoms had been so severe that he had to be hospitalised.

The patient presented with a temperature of 39.2 C. He was tachycardic (104 bpm), his respiratory rate was 16 breaths per minute, he was hypertensive (131/53 mmHg), and his saturations were 97% on room air. Cardiovascular and chest examinations were unremarkable. There was no lymphadenopathy, and his abdomen was soft with mild right upper quadrant tenderness. He had no skin rashes, history of nausea, vomiting, diarrhoea, or loose stool. He had not been vaccinated prior to his holiday but had been exposed to insect bites and unfiltered water there.

Admission bloods revealed a normal bone profile and U&Es, but deranged liver function tests, with an ALT of 351 U/L (normal range: 10–50 U/L) and AST of 88 IU/L (normal range: 0–40 IU/L). His C-reactive protein was also high, at 45.4 mg/L (normal range: <5 mg/L).

Blood tests for antibodies against the gastric parietal smooth muscle, liver, and kidneys came back negative, as did the antinuclear antibody (ANA) and antineutrophil cytoplasmic antibody (ANCA) screens. Regarding viral screens, he was IgG positive for both the Epstein–Barr virus and cytomegalovirus virus, but negative for IgM for both. There were negative antibody tests for malaria, typhus, glandular fever, dengue, HIV, syphilis, rickettsial disease, chikungunya, and leptospirosis. Total antibodies for hepatitis A were positive, but IgM was negative. The hepatitis B core antibodies were nonreactive, and the antibodies for hepatitis C were negative.

An ultrasound of the liver showed a slight prominence of the intrahepatic duct, possibly due to recent hepatitis. A CT scan of the head was normal, and an MRI was unremarkable.

He was started on ciprofloxacin but then switched to 2 g of cefuroxime and doxycycline.

Further investigations for infection were carried out, including midstream specimen of urine (MSU) and stool microbiology, culture, and specimen (MC&S), but these all came back normal.

A lumbar puncture showed less than one white cell, less than one red cell, and negative Gram stain. PCR for herpes simplex virus (HSV) and VCP were also negative.

Three days later, the patient became emotionally labile and confused and started presenting with acute paranoid psychotic symptoms. A psychiatric liaison referral was thus made from the treating physician in the general hospital (the third author) to the corresponding author.

On mental state examination, the patient had a flat affect and subjectively low mood, anhedonia, impaired concentration, and reduced appetite. He further stated that he felt suicidal, but no specific plans were described.

A collateral history from his wife highlighted that these symptoms were completely out of character for him. He had no personal history of psychiatric illness besides professional burnout three years previously and was otherwise fit and well. He reported that he had not taken any psychotropic drugs and drank alcohol minimally.

Repeat blood testing showed raised liver function tests: his peak AST was 242 IU/L and his peak ALT was 490 IU/L. He was thus seen by an infectious disease specialist and switched to the broad-spectrum antibiotic, azithromycin.

Subsequently, porphyria screening and urine toxicology (for psychotropic drugs including benzodiazepines, cannabis, cocaine, amphetamine, and opiates) came back negative. Screening for heavy metals came back negative too, except for mercury, which was slightly elevated at 29 nmol/L. Notably, the urine mercury was 0.9 nmol/L and the mercury-to-creatinine ratio was 0.41 (normal range <5.5). Anti-NMDA-receptor antibodies came back negative.

Microbiology tests came back negative, with the notable exception of *Brucella*. Repeat serology confirmed the brucellosis infection. The patient was then started on 600 mg rifampicin daily, as well as 100 mg doxycycline twice daily.

Within one day, the patient's psychotic symptoms disappeared. Ten days following admission, he was discharged and referred for outpatient counselling to process his acute hospitalisation. He has been asymptomatic subsequently.

## 3. Discussion and Conclusions

The patient's fever, abdominal pain, and constipation suggest foodborne exposure to *Brucella*, although it could also have been acquired via contact with wild animals on the patient's safari trip (during his holiday).

It is striking that the majority of laboratory tests, including MCU and MC&S, came back negative. The ALT, AST, CRP, and ESR levels, coupled with liver enlargement and prominent intrahepatic ducts, point towards localised infection in the liver. Given the psychosis, the second site of infection appears to be the brain, although again it is striking (but not unusual) that the CSF screen was negative.

Hepatomegaly is a less frequent sign of *Brucella* and suggests a rarer form of brucellosis, further supported by the rare onset of psychiatric symptoms later in the clinical presentation. There is no clearly established mechanism for *Brucella*-induced psychosis, but several suggestions have been put forward within the literature [14–16].

One such hypothesis is that the body's response to infection or autoimmunity may lead to defective neurological pathways and consequently psychosis. This arises from the long-standing observation that congenital and adult infections alike, as well as autoimmune disorders, have been linked to psychotic disorders [14].

Possible mechanisms include necrotic death of macrophages, the prevention of microglial apoptosis, and downregulation of MHC1 in microglia; interference with additional neurological functions of MHC1; [14] and involvement of *Brucella*-induced cytokines and matrix metalloproteinases (MMPs) [15, 16].

Astrocytes are crucial in regulating neuronal synapses in the brain [17], and NMDA receptors are already established to be involved with psychosis. One MMP, MMP-9, can influence the synaptic glutamic N-methyl-D-aspartic acid (NMDA) receptor, the inhibition of which has already been linked to psychosis, as in the case of anti-NMDA antibody-mediated psychosis; studies have shown that *Brucella* can produce MMP-9 via infected astrocytes in the central nervous system [18].

Another hypothesis is that by invading and killing brain cells in certain regions, *Brucella* is able to interfere with normal neuronal pathways. The hippocampus, implicated as a locus of pathology in psychosis [18], is one such potential target region.

In other infection-induced psychoses, the production of autoantibodies has been noted [19]. However, this appears to be a less likely cause of psychosis in this patient, given the short timescale in which his symptoms resided once *Brucella* had been eradicated.

For similar reasons, interference with genes seems less likely to be the mechanism of psychosis. However, it is possible that genetic predispositions, such as polymorphisms of the DRD2 and DRD4 genes (which code for dopamine receptors 2 and 4, respectively), a nucleotide repeat sequence error in the GNAL gene (which codes for the G-protein alpha subunit normally coupled to the D1 dopamine receptor) [20], and genetic mutations in the MHC locus [21], made the patient more vulnerable to the symptoms he experienced.

This case highlights the importance of considering brucellosis as a differential diagnosis of acute and atypical psychoses. The exact mechanism of *Brucella*-induced psychosis remains unclear, although the psychotic symptoms can resolve quickly with appropriate antibiotic treatment, in keeping with other similar case reports of neurobrucellosis [6, 13].

## Data Availability

Data used to support this study were obtained from patient records at Princess Grace Hospital.

## Abbreviations

ALT: Alanine aminotransferase
ANA: Anti-nuclear antibodies
ANCA: Antineutrophil cytoplasmic antibodies
AST: Aspartate aminotransferase
CNS: Central nervous system
CRP: C-reactive protein
CSF: Cerebrospinal fluid
CT: Computed topography
DRD2: Dopamine receptor 2
DRD4: Dopamine receptor 4
ESR: Erythrocyte sedimentation rate
GNAL: G-protein subunit alpha L
HIV: Human immunodeficiency virus
HSV: Herpes simplex virus
LSD: Lysergic acid diethylamide
MC&S: Microbiology, culture, and specimen
MHC1: Major histocompatibility complex 1
MMP: Matrix metalloproteinase
MRI: Magnetic resonance imaging
MSU: Midstream specimen of urine
NMDA: N-Methyl-D-aspartate
PCR: Polymerase chain reaction
U&Es: Urea and electrolytes
VCP: Valosin-containing protein.
